# Comparative Transcriptome Analysis Identified Key Pathways and Genes Regulating Differentiated Stigma Color in Melon (*Cucumis melo* L.)

**DOI:** 10.3390/ijms23126721

**Published:** 2022-06-16

**Authors:** Yuanzuo Lv, Sikandar Amanullah, Shi Liu, Chen Zhang, Hongyu Liu, Zicheng Zhu, Xian Zhang, Peng Gao, Feishi Luan

**Affiliations:** 1 Key Laboratory of Biology and Genetic Improvement of Horticulture Crops (Northeast Region), Ministry of Agriculture and Rural Affairs, Northeast Agricultural University, Harbin 150030, China; lvyuanzuo@126.com (Y.L.); sikandaraman@yahoo.com (S.A.); shiliu@neau.edu.cn (S.L.); wkzc351504709@126.com (C.Z.); hyliu@neau.edu.cn (H.L.); zzc1983sc@163.com (Z.Z.); 2 College of Horticulture and Landscape Architecture, Northeast Agricultural University, Harbin 150030, China; 3 Horticulture College of Northwest A&F University, Yangling, Xianyang 712100, China; zhangxian@nwsuaf.edu.cn

**Keywords:** melon, stigma color, chlorophyll, RNA-seq

## Abstract

Stigma color is an important morphological trait in many flowering plants. Visual observations in different field experiments have shown that a green stigma in melons is more attractive to natural pollinators than a yellow one. In the current study, we evaluated the characterization of two contrasted melon lines (MR-1 with a green stigma and M4-7 with a yellow stigma). Endogenous quantification showed that the chlorophyll and carotenoid content in the MR-1 stigmas was higher compared to the M4-7 stigmas. The primary differences in the chloroplast ultrastructure at different developmental stages depicted that the stigmas of both melon lines were mainly enriched with granum, plastoglobulus, and starch grains. Further, comparative transcriptomic analysis was performed to identify the candidate pathways and genes regulating melon stigma color during key developmental stages (S1–S3). The obtained results indicated similar biological processes involved in the three stages, but major differences were observed in light reactions and chloroplast pathways. The weighted gene co-expression network analysis (WGCNA) of differentially expressed genes (DEGs) uncovered a “black” network module (655 out of 5302 genes), mainly corresponding to light reactions, light harvesting, the chlorophyll metabolic process, and the chlorophyll biosynthetic process, and exhibited a significant contribution to stigma color. Overall, the expression of five key genes of the chlorophyll synthesis pathway—*CAO* (*MELO03C010624*), *CHLH* (*MELO03C007233*), *CRD* (*MELO03C026802*), *HEMA* (*MELO03C011113*), *POR* (*MELO03C016714)*—were checked at different stages of stigma development in both melon lines using quantitative real time polymerase chain reaction (qRT-PCR). The results exhibited that the expression of these genes gradually increased during the stigma development of the MR-1 line but decreased in the M4-7 line at S2. In addition, the expression trends in different stages were the same as RNA-seq, indicating data accuracy. To sum up, our research reveals an in-depth molecular mechanism of stigma coloration and suggests that chlorophyll and related biological activity play an important role in differentiating melon stigma color.

## 1. Introduction

Flower pollination is a critical stage during reproduction in flowering plants, where the male gamete (pollen grains) from the anther comes into contact with the female gamete (stigma), and successful reproduction is highly dependent on efficient pollinators [1]. The pollinators are usually described either as an abiotic factor (wind) or a biotic factor (insect) [2]. The distinction in floral color patterns at the center of the flower directs the pollinators in finding the desired nectars [3,4], and these patterns are known to be effective in handling different photoreceptor classes, e.g., blue, green, and UV ranges.

Each crop plant bears different floral morphology, and some studies have explained the complexity and diversity of the different substances involved in determining stigma colors. In *Crocus sativus*, the transformation of an undeveloped yellow to a fully developed red stigma is affected by the accumulation of zeaxanthin and phytoene desaturase as well as the massive accumulation of *CsBCH* and *CsZCD* transcripts [5]. The significant accumulation of naringenin chalcone developed a mutant-type yellow stigma (*ys*) that was different from the stigma of wild-type tomatoes [6]. The candidate gene controlling stigma color has been widely reported in rice. The first gene controlling rice stigma color was identified by Oka (1991). Later, *Ps-5* was isolated from the ‘Nipponbare’ mutant with purple stigmas, and a single recessive gene was located on Chromosome 8 [7]. In rice, anthocyanin accumulation in the stigma was determined by both *DFR* and *OsC1*, which triggered a purple color [8]. In a recent study, genetic analysis showed that a major gene controlling the color of melon stigma is located on Chromosome 8 and was mapped to Chromosome 8 within a 268 kb interval [1].

Melon (*Cucumis melo*, 2n = 24) is considered an important economic crop of the Cucurbitaceae family, which exhibits broad phenotypic diversity. In 2020, China alone produced 13,838,234 tonnes of melon at a harvested area of 385,756 hectares (ha) (FAOSTAT; http://faostat.fao.org (accessed on 2 December 2021)). In Cucurbit crop plants, cucumber and watermelon exhibit yellow stigmas under natural conditions, but some varieties of melon exhibit yellow stigmas, and others are green. Chlorophyll (Chl) has unique and essential roles in photosynthetic light-harvesting and energy transduction, but its biosynthesis, accumulation, and degradation are also associated with chloroplast development, photomorphogenesis, and chloroplast-nuclear signaling [9,10]. Chlorophyll is widely present in plant leaves and fruits, but it has not been reported that chlorophyll can affect plant stigma color [11,12,13].

In recent years, next-generation deep sequencing has provided new methods of transcriptome analysis, termed as RNA sequencing (RNA-Seq). This sequencing approach provides a precise measurement for transcript levels to reveal the response mechanisms towards specific stimuli [14]. In the last decade, this approach has been widely used for an in-depth molecular understanding of various adaptive evolutions, the host immune system, and stress responses in many living organisms [15], e.g., rainbow trout (*Salmo gairdneri*) [16], fish species [17], sea bream (*Sparu aurata*) [18], sea bass (*Lateolabrax japonicas*) [19], and large yellow croaker (*Larimichthy crocea*) [20]. RNA-seq has been similarly used for a comparative transcriptomic analysis of the morphology and significant, quality-related traits of cucurbit fruit crops, e.g., the climacteric behavior and hardness of watermelon flesh in relation to fruit ripening (*Citrulus lanatus* L.) [14,21], and the powdery mildew resistance mechanism in melon (*Cucumis melo* L.) [22].

Differentially expressed genes (DEGs) involved in various biological processes can be identified by RNA-seq. The elucidation of these changes at the transcriptome level would facilitate our in-depth understanding of key biological and physiological mechanisms [23]. These studies also identified candidate regulatory pathways involved in the response to heat stress, including metabolism, protein folding and degradation, and immune response, indicating that these biological pathways are critical for regulating significant traits. Further, weighted gene co-expression network analysis (WGCNA) is a method frequently used to explore the complex relationships between genes and phenotypes. WGCNA has been widely used to analyze high-throughput sequencing data [24], which has aided in the identification of key genes involved in the development of peculiar traits [25].

Herein, we performed transcriptome analysis between two contrasted melon lines, MR-1 (green stigma) and M4-7 (yellow stigma), during three key developmental stages. Endogenous chlorophyll and carotenoid content was quantified in the stigmas of both melon lines, and differentiation in the chloroplast ultrastructure was also observed. Moreover, comparative transcriptomes of the stigmas of both melon lines were performed, and WGCNA presented the key pathway and DEGs involved in the formation of colors in melon stigmas. The relative expressions of candidate identified genes were checked using quantitative real time polymerase chain reaction (qRT-PCR) and validated with RNA-seq data. We believe that our research findings would enrich any further genetic and genomic resource studies of stigma color formation in numerous crop plants.

## 2. Results

### 2.1. Pigment Content and the Structural Basis of the Stigma

**Figure 1:**
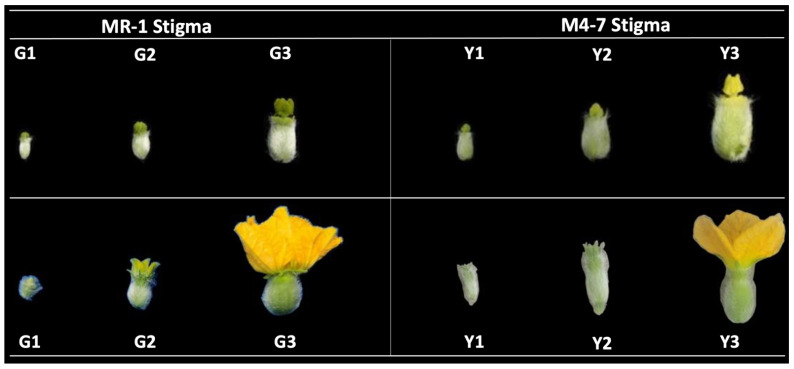
A pictorial view of contrasted melon stigmas (green: “G”; yellow: “Y”) at three key developmental stages.

**Figure 2:**
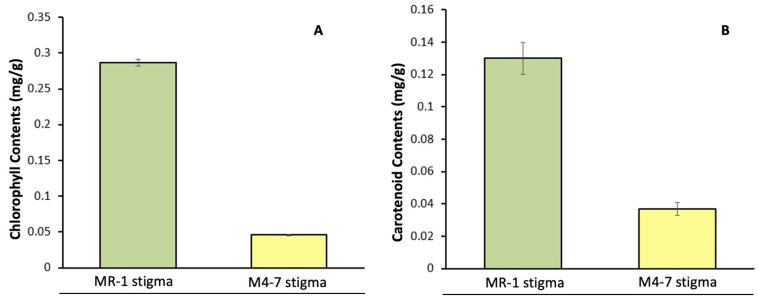
Endogenous pigment quantification in the stigmas of two contrasted melon lines. (**A**) Chlorophyll content; (**B**) carotenoid content.

Visual flower inspection showed that the stigma of MR-1 was green from S1 to S3; however, the stigma of M4-7 was green from S1 to S2 stages and gradually became yellow from S2 to S3 stages (Figure 1). The endogenous chlorophyll content was checked in the stigmas of MR-1 and M4-7 during S3 stage (Figure 2A), which demonstrated a high chlorophyll content (0.29 ± 0.004 mg/g) in the MR-1 line compared to the M4-7 line (0.03 ± 0.001 mg/g). The carotenoid content of the MR-1 line remained higher (0.28 ± 0.01 mg/g) than in the M4-7 line (0.04 ± 0.004 mg/g) (Figure 2B), but the carotenoid/chlorophyll ratio was much lower (0.07) in MR-1 than in M4-7 (2.33). This primary difference may have caused the yellow stigma of M4-7, even though it contained less carotenoid content.

**Figure 3:**
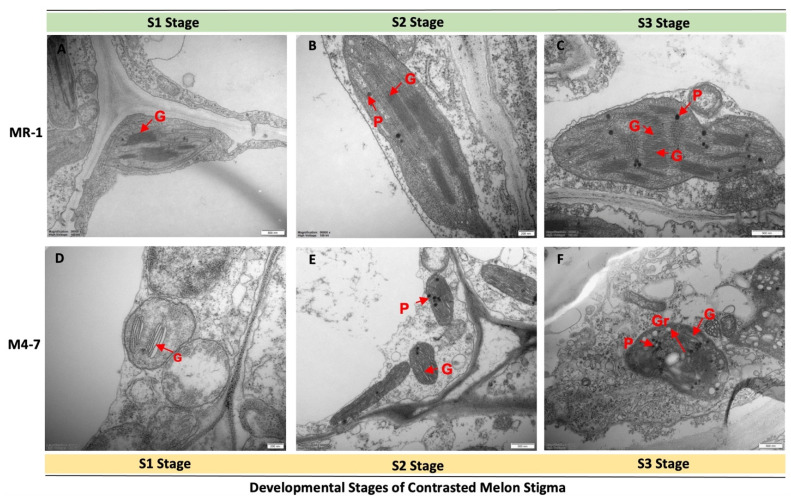
Primary differentiation in chloroplast ultrastructure of the two contrasted melon lines during three developmental stages (S1, S2, and S3). (**A**–**C**) The structural variation in the chloroplast of the MR-1 line and (**D**–**F**) the M4-7 line. G: granum; P: plastoglobulus; Gr: starch grains.

The internal ultrastructures of both melon stigmas were observed by transmission electron microscopy (Figure 3). The results showed that the chloroplasts of both materials began to differentiate during S1 stage, but the granum in MR-1 was higher in quantity and was well-developed. At S2 stage, the amount of MR-1 granum remained significantly higher than M4-7; however, a small number of starch grains were observed in M4-7. The increase in granum during the two stages was consistent with the stigma of MR-1 and M4-7, exhibiting a green stigma.

At S3 stage, the MR-1 chloroplast structure exhibited the highest amount of granum, along with plastoglobulus. In contrast, the M4-7 chloroplast structure showed a breakdown of granum, with a high production of plastoglobulus and starch grains, and the color of the stigma completely changed to yellow. In general, microscopic observations were consistent with the changes in stigma color. Therefore, we suggest that differences in chloroplasts provide the structural basis for differences in stigma color.

### 2.2. Overview of Transcriptomic Data

**Figure 4:**
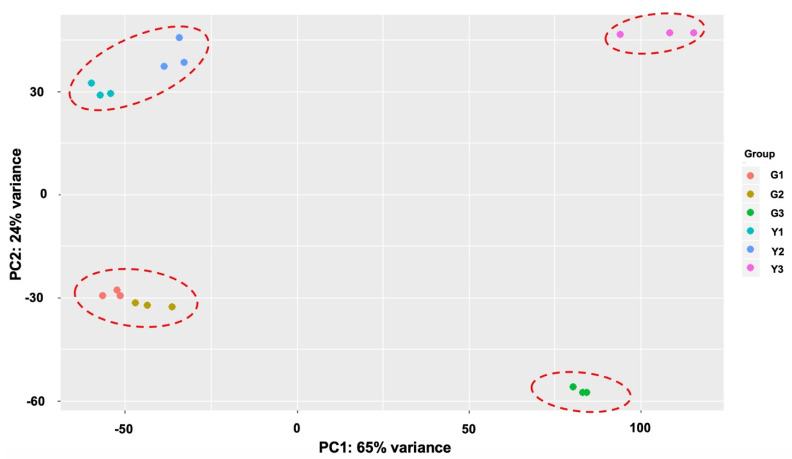
PCA biplot showing transcriptome clusters of the developmental stages of the different stigmas of MR-1 and M4-7.

To investigate the major differences in the transcriptomic dynamics during the stigma developmental stages of the MR-1 and M4-7 lines, we performed principal component analysis (PCA) based on average FPKM values (Figure 4). The stigma transcriptome of both melon lines exhibited similar function and activity at S1 stage (G1 and Y1) and S2 stage (G2 and Y2), but substantial differences at S3 stage (G3 and Y3) were subsequently observed. Mainly, the first two stages (S1 and S2) of both melon lines exhibited a closer relationship with each other, and G1G2 and Y1Y2 can be seen in one cluster, respectively. However, the G3 and Y3 of S3 stage were highly inconsistent with the first two stages (S1 and S2). The findings of PCA analysis showed a consistent trend with the color transformation process of the melon stigma. Therefore, we believe that differences in the transcriptional level at S3 determined the developmental specificities and stigma color of the contrasted melon lines (MR-1 and M4-7).

### 2.3. Identification of DEGs during Stigma Development Stages

To identify the DEGs of the melon stigma color, we compared the FPKM values of each gene in both lines, MR-1 and M4-7, during different developmental stages. DEGs were retained with a fold change of >2, and false discovery rate (FDR) correction was set at *p* < 0.01. The comparative transcriptome data were analyzed at different developmental stages, where both lines showed a consistent trend during the first two stages (S1 and S2) and a significant increase in DEGs was observed at S3 (Figure S1). We also compared the transcriptome data of the same stage for both varieties and observed that the number of DEGs was similar at S1 and S2 stages but doubled at S3 stage (G1 vs. Y1: 2523; G2 vs. Y2: 2614; G3 vs. Y3: 5312) (Figure S2). However, the total DEGs of S3 stage seemed more likely to be associated with melon stigma color.

**Figure 5:**
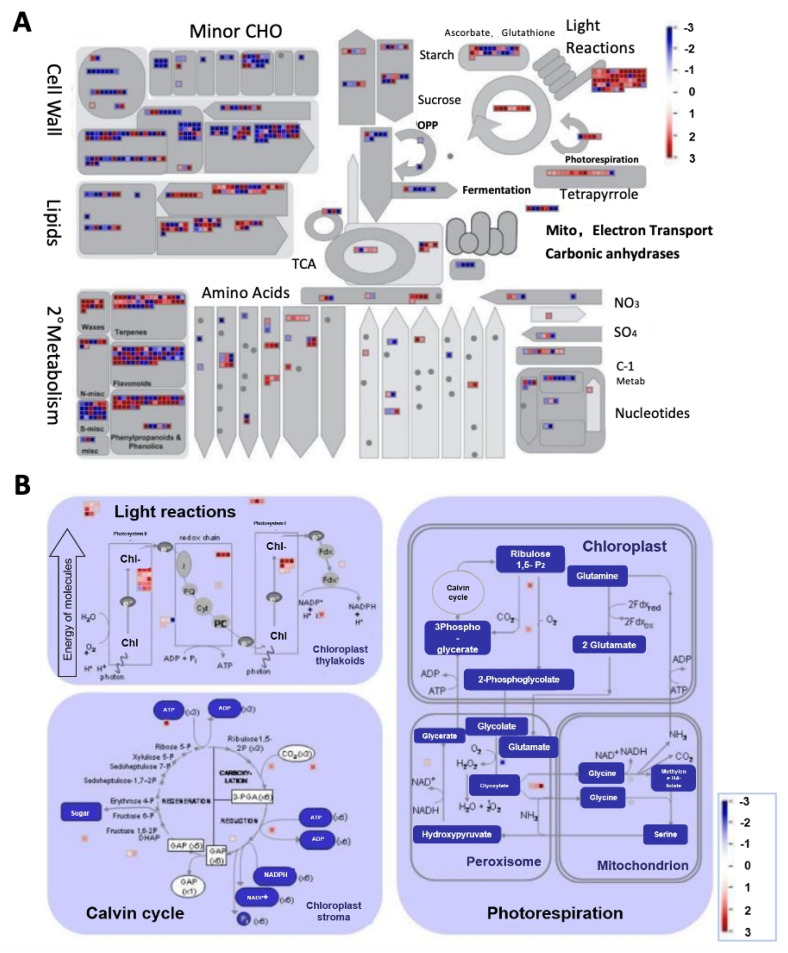
MapMan overview of the metabolic pathways (**A**) and photosynthesis (**B**) with the differential expression profile in MR-1 as compared to M4-7 during S3. Differentially expressed genes (DEGs) between MR-1 and M4-7 at S3 were loaded into MapMan to generate the overview. On the log2 scale, dark blue and dark red represent higher and lower expressions in MR-1 compared to M4-7, respectively.

The regulatory network involved in plant development is very complex and involves various aspects of biological processes. To further illustrate the key biological functions of the transcriptome at different stages of MR-1 and M4-7, we conducted gene ontology (GO) enrichment analysis of all DEGs during all stages. It was noticed that the identified DEGs of both melon lines were mainly enriched in many of the same terms, e.g., plant organ morphogenesis (GO term: 1905392), response to auxin (GO term: 0009733), and regulation of hormone levels (GO term: 0010817) (Table 1). Overall, the biological processes involved in melon stigma development were consistent, despite the color differences. It is noteworthy that chlorophyll and the related biological pathways (photosynthesis and chloroplast localization) were enriched in S2 and S3 only. Therefore, we visualized the expression profiles of DEGs between MR-1 and M4-7 at S3 using MapMan (Figure 5A). The highest number of DEGs in the light reaction pathway and further analysis revealed that the transcriptional activity of the MR-1 line was higher than that of the M4-7 line, in terms of the Calvin cycle and the light reaction (Figure 5B).

### 2.4. Identification of WGCNA Modules Associated with Photosynthetic Reactions

**Figure 6:**
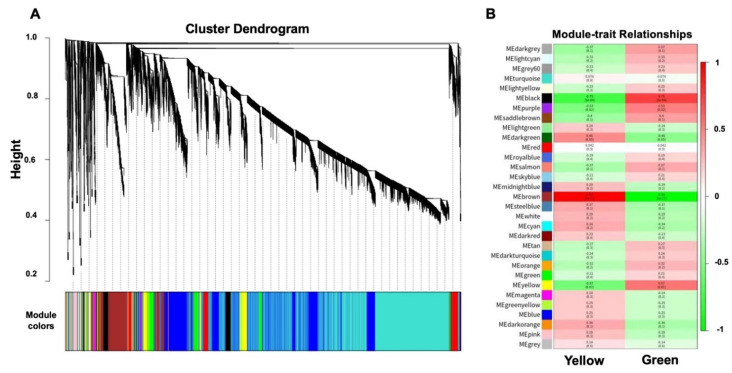
The weighted gene co-expression network analysis (WGCNA) of identified DEGs during the three developmental stages of MR-1 and M4-7. (**A**) Hierarchical cluster tree showing 31 modules of co-expressed genes, where each DEs is represented by a leaf in the tree, and each of the 31 modules is represented by a major tree branch. The lower panel shows modules in designated colors, such as yellow and black. (**B**) The module of the green stigma exhibits correlations and *p*-values (in parentheses).

**Figure 7:**
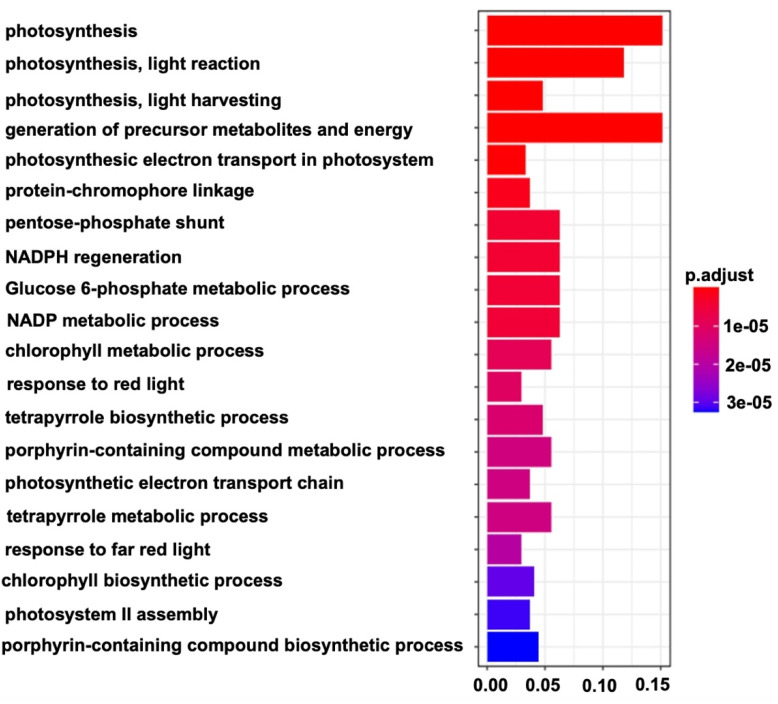
Significant GO terms enriched in the DEGs of the ‘black’ module between the MR-1 and M4-7 stigmas.

The co-expression networks were constructed on the basis of pairwise correlations of gene expression across all samples. We identified 31 modules by weighted gene co-expression network analysis (WGCNA) as shown in the constructed dendrogram (Figure 6A). The analysis of module–trait relationships revealed a “black” network module (655 out of 5302 genes), which was highly correlated with a green stigma (Figure 6B). Gene ontology (GO) enrichment of genes in the black module significantly depicted photosynthesis, light harvesting, a generation of precursor metabolites, and a chlorophyll biosynthetic process (Figure 7).

**Figure 8:**
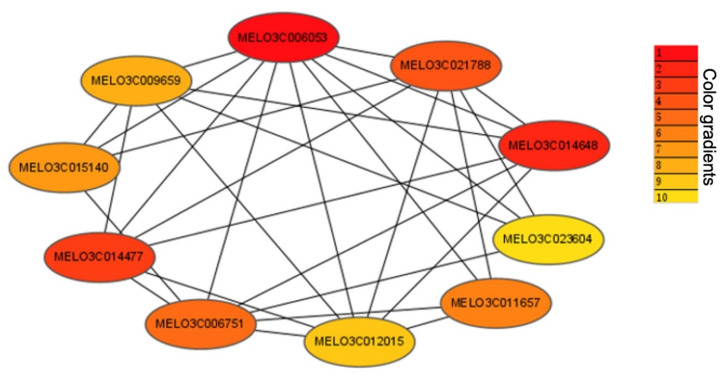
Top 10 hub gene-related networks with weight values in the “black” module. The red color density indicates a higher connectivity, as compared to the light color density.

WGCNA can also be employed to construct the gene networks, in which each node represents a hub gene, and connecting lines between hub genes represent the co-expression of correlations. Here, we only show the top ten hub genes for ‘radiality’ values (Figure 8). Mainly, four of these hub genes (*MELO03C011657*, *MELO03C014648*, *MELO03C021788*, and *MELO03C006053*) are annotated as chlorophyll-related (Table 2).

**Figure 9:**
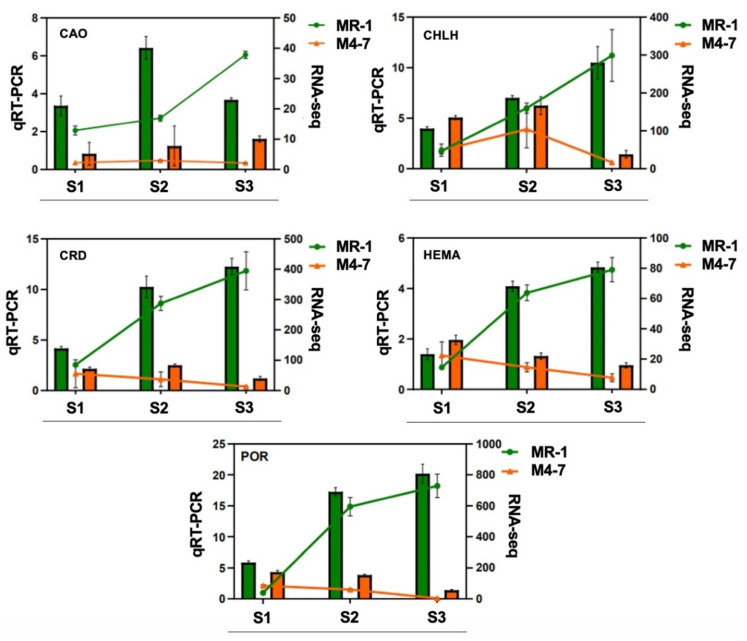
Different expression levels of five key genes of the chlorophyll synthesis pathway at different stages of stigma development in MR-1 and M4-7; bar graphs indicate qRT-PCR results and line graphs indicate RNAseq results. *CAO*: *MELO03C010624*; *CHLH*: *MELO03C007233*; *CRD*: *MELO03C026802*; *HEMA*: *MELO03C011113*; *POR*: *MELO03C016714.*

Therefore, we further determined the expression of five key genes of the chlorophyll synthesis pathway in MR-1 and M4-7 at different stages of stigma development by performing qRT-PCR. The results are consistent with our prediction that the expression of these genes gradually increases during the stigma development of MR-1 and decreases in M4-7 at S2 (Figure 9). In addition, the expression trends in different stages were similar to RNA-seq, indicating the accuracy of the RNA data.

## 3. Discussion

### 3.1. Differences in Chloroplast Development of the Contrasted Melon Lines (MR-1 and M4-7)

Color phenotypes are often considered a communication signal in plants whose genetic diversity is driven by the direct natural selection of biochemical (pigmentation) and structural (cell shape) characteristics, resulting in color. At the physiological level, pigment content and chloroplast development were associated with coloration in higher plants [10]. The central accumulation of photosynthesis takes place in the chloroplast, which is composed of a chloroplast and a thylakoid membrane matrix (platform for photosynthesis’s light reactions) [26].

Herein, transmission electron microscopic observations revealed a large difference in the process of chloroplast development between different stages of contrasted melon lines (MR-1 and M4-7). Some studies have reported that differences in chloroplast development affect chlorophyll content and thus affect leaf coloration [27,28,29,30]. It was reported that a tea cultivar with a yellow leaf exhibits a lack of chlorophyll content due to abnormal chloroplast structures under high light intensity; however, the DEGs were supposed to be predominantly involved in chloroplast development and photosynthetic pigment synthesis [31]. RNA-seq analysis revealed that the chloroplast ultrastructure and multiple pigment biosynthesis have a significant impact on leaf color formation in mutants of *Anthurium andraeanum* ‘Sonate’ [10], and nitrogen deficiency also triggers irregular chloroplast arrangement in rice leaves due to the gradual development of unbalanced thylakoids [32]. In our study, the number of cystoids in MR-1 chloroplasts, compared to M4-7 chloroplasts, was found to be excessive from S1 to S3; however, the cystoid membrane was clearer, and granules were more uniform. Carotenoid accumulation in the cystoid membrane was higher in MR-1 than in M4-7 [33,34]. Carotenoids are potent antioxidants that act as an effective barrier to high-energy blue light [35,36], so the higher carotenoid content of the MR-1 stigma may help its female flowers to be able to withstand stronger light. Similarly, in the rice mutant (*CH1*), abnormal chloroplast development along with thinner grana was found to be associated with a reduction in pigmentation, an elevated Chl a/b, and reduced photosynthetic capacity [37], but further experiments are needed to demonstrate this assumption.

### 3.2. A Complex Regulatory Network Exists for Melon Stigma Color Development

Transcriptome analysis has evidently shown changes in gene expression, which helped to gain insight into biological processes and revealed spatiotemporal expression patterns across the genome [30]. In this study, the transcriptomes of two melon lines (MR-1 with a green stigma and M4-7 with a yellow stigma) were characterized to identify the candidate DEGs involved in three stages (S1, S2, and S3). It was found that the number of DEGs of S1 and S2 was lower than that of S3 (Figures S1 and S2), and PCA analysis similarly showed a consistent trend with the color transformation process of contrasted melon stigma (Figure 4). Therefore, it is evidently assumed that the difference in the transcriptional level at S3 may determine the developmental specificities and stigma color of the MR-1 and M4-7 lines.

It has been stated that many metabolites with complex regulatory networks play simultaneous roles in the process of plant growth and development [38]. For instance, phenylpropanoid and lignin pathways are significantly involved in the transition from pit-hardening to the cell enlargement stage and subsequently to the ripening stage in peaches [39]. In this study, GO enrichment analysis presented several important biological processes, cellular and metabolic processes, DNA binding, oxidoreductase activity, transcription regulation, and nucleus and cell wall activities associated with melon stigma development during S1 and S2 stages. These results suggest that the biological processes involved in the development of melon stigma were irrespective of the stigma color. Similar phenomena were observed in peach and olive flesh, where carotenoids accumulated without any noticeable changes in the early stages but with a significant accumulation during the ripening period [40,41].

### 3.3. An Important Biological Process Played a Key Role in Chlorophyll Synthesis Variation and the Formation of Different Colors in Melon Stigmas

The WGCNA of 5302 DEGs enabled us to identify the “black” module that includes 655 genes, which were highly correlated with stigma color. According to the GO enrichment analysis of the “black” module, the critical stage (S3) of color presentation showed a maximum number of expressed genes related to Photosystem I, Photosystem II, chloroplast, photosynthesis, and light reactions. This illustrated the key role of chlorophyll in the color presentation of melon stigmas. Thus, the WGCNA analysis package [42,43] has been extensively used for similar analyses in many studies [44,45,46,47], and it was also meaningful in the biological sense of our study.

Chlorophyll and carotenoids are the main pigments contributing to significant color formation [48]. It was reported that different compositions and different concentrations of chlorophyll and carotenoids result in a variety of colors, e.g., red, orange, yellow, green, light green, and white, in the flesh of Cucurbit fruits [49,50]. In this study, we further examined the endogenous chlorophyll and carotenoid content of two contrasted melon lines and observed the metabolic characteristics of the varying stigma coloration. The endogenous chlorophyll content of the green stigma of MR-1 was much higher compared to that of the yellow stigma of M4-7, and this result is consistent with the characteristics of the transcriptome data. In addition, the carotenoid content of the green stigma of MR-1 was higher than that of M4-7, but the overall carotenoid content of both materials was small, and we speculated a limited role in stigma coloration. There are other studies with similar results, which indicate that chlorophyll-deficient mutant tomatoes turned yellow due to abnormal chloroplast development [13]; however, yellow naringenin chalcone and carotenoids clearly exist in the peels of dark-green fruits [51].

In this study, four of the 10 candidate genes were obtained by WGCNA analysis, and they were directly related to the chlorophyll content. The differential roles of these genes have been reported in a few published studies. The *MELO3C006053* gene encodes curvature thylakoid 1 (*CURT1*), which is a major contributor for the internal shaping of the chloroplast [52]. In *Arabidopsis thaliana*, CURT1 proteins are present in etioplasts, and a loss of CURT1 results in the looser packing of the PLB paracrystalline lattice, which leads to a faster disassembly of PLBs, reduces chlorophyll synthesis, and triggers the accumulation of LHCII [53]. The *MELO3C021788* gene was annotated as the Photosystem I (*PSI*) reaction center, which is a large super-complex protein that catalyzes the light-dependent oxidation of plastocyanin and reduces the ferredoxin; however, its biogenesis process requires a specific regulatory and quality control network [54]. The *MELO3C014648* gene encodes a plastid lipid-associated protein (PAP), which belongs to the fibrillin family related to chromoplast fibrils, thylakoids, and photosynthetic antenna complex [55]. This PAP protein has been reported to perform an important role in the development of chloroplasts in watermelon and is similarly involved in the chlorophyll metabolic pathway, regulating the pale green flesh color due to the maximum endogenous chlorophyll accumulation [56,57]. The *MELO3C011657* encodes the tetrapyrrole-binding protein, which has been significantly demonstrated in the *Arabidopsis papp5* mutant, and it has been shown that the tetrapyrrole-binding protein is mainly associated with PAPP5 in complex components involved in the downstream of the plastid signaling pathway of tetrapyrrole Mg-ProtoIX/Mg-ProtoIX-ME [58]. The complexity of these components can act as a negative regulator of PhANG expression during chloroplast development, which ultimately affects the chloroplast morphology associated with plant flower color morphology [59]. Most importantly, we believe that our current research findings demonstrate comprehensive bio-information about the candidate pathways and associated genes involved in the determination of melon stigma color. 

## 4. Materials and Methods

### 4.1. Plant Materials and Sampling Stages

The seeds of contrasted melon lines, MR-1 with a green stigma and M4-7 with a yellow stigma, were obtained from the Laboratory of Molecular Genetics and Breeding in Watermelon and Melon, at Northeast Agricultural University, Harbin, China. Thirty plants of each melon line were cultivated in a plastic greenhouse at Xiangyang Agricultural Experiment Station. The plant developmental stages were regularly checked, and stigmas of both melon lines (MR-1 and M4-7) were collected during the three key developmental stages, including the first day (the S1 stage, where stigmas of both melon lines were green), the seventh day (the S2 stage, where the stigmas of MR-1 were green, but the stigmas of M4-7 ranged from green to partially yellow), and the twelfth day (the S3 stage, where the stigmas of MR-1 were still green, but the stigmas of M4-7 were completely yellow), respectively (Figure 1). The required amount of samples was collected and used for RNA sequencing analysis and other subsequent experimentation.

### 4.2. Metabolite Detection and Transmission Electron Microscopy

The collected stigmas of both melon lines were subjected for total carotenoid profiling using the Plant Carotenoid ELISA Kit (Guiechem) and a microplate reader. A sufficient quantity of stigmas (0.1 g) was taken from the S3 stage (with completely differentiated stigma colors) of both melon lines, and samples were then immersed in 0.9 mL of lysate and homogenized with a bullet blender tissue homogenizer for 5 min. The final solution was later centrifuged at 5000× rpm for 5 min, and the upper supernatant was removed for subsequent testing. The absorption rate of the final extracts was measured at a 450 nm wavelength using a microplate reader, and carotenoids were determined. 

For endogenous chlorophyll quantification, 0.1 g of stigmas were cut from both melon lines, immersed in 1 mL of acetone (80%, *v*/*v*), and homogenized using a bullet blender tissue homogenizer for 5 min. The absorption of chlorophyll extracts was measured at 663.6 and 646.6 nm wavelengths using a Hitachi U2800 microplate reader, and chlorophyll was determined according to a previously reported method [60]. Similarly, the collected stigmas of three key stages were subsequently checked to observe the major differentiation in chloroplast ultrastructure with a transmission electron microscope, according to a previously reported method [61].

### 4.3. RNA Sequencing and Transcriptomic Analysis

The stigmas of both melon lines were collected at all three stages. Samples were quickly frozen in cryogenic liquid nitrogen and stored at an ultra-low temperature of −80 °C until further experimentation. The RNA-seq libraries were produced with 4 μg of total isolated RNA following the instructions of the Illumina TruSeq RNA sample preparation kit (FC-122-1001). After amplification and purification, the libraries with an average size of 300 bp were checked on a 2% low-range ultra-agarose gel (BIO-RAD). RNA quality (RIN > 8) and library size were assayed on a 2100 Bioanalyzer (Agilent Technologies). The RNA-seq library construction, Illumina sequencing, and read mapping were performed at Novogene (Beijing, China, https://www.novogene.com/, accessed on 18 May 2022). 

The quality of the raw sequenced reads from all samples was checked using FastQC (v0.11.2). All clean-end reads obtained from each sample were then mapped onto the melon reference genome (DHL92, v3.6.1) using the default parameter setting of TopHat (v2.0.11). The unique mapped reads of each specific transcript were counted using HTSeq (v0.6.1). The raw RNA-seq data were uploaded to the Sequence Read Archive (SRA) database (PRJNA839169, 18 independent libraries including biological replicates in this bioproject) for access by the scientific community (https://www.ncbi.nlm.nih.gov/bioproject/?term=PRJNA839169, accessed on 18 May 2022).

The gene abundances were calculated and normalized to RPKM (reads per kb per million reads). The edgeR package (http://www.rproject.org/, accessed on 18 May 2022) was used to identify the differentially expressed genes (DEGs) across the contrasted groups with fold changes ≥2 and a false discovery rate (FDR) <0.05. Pearson’s correlation coefficients between the independent biological replicates for each sample were calculated and demonstrated using the R package (heatmap). DEGs were then subjected to an enrichment analysis of GO (gene ontology) pathways, gene numbers were calculated for every term, and significantly enriched GO terms in DEGs compared to the genome background were defined by a hypergeometric test. The calculated *p*-value was subject to FDR correction, taking an FDR of ≤0.05 as a threshold.

The co-expression networks were constructed using the weighted gene co-expression network analysis (WGCNA, v1.47) package in the R language programming tool (v4.2.0) [46]. After filtering the genes, gene expression values were imported into the WGCNA to construct co-expression modules. Expression correlation coefficients of the remaining genes were then calculated to search a suitable soft threshold for building gene networks using a scale-free topology model [62]. To identify biologically significant modules, module engines were then used to calculate the correlation coefficients. The intramodular connectivity (function soft connectivity) of each gene was calculated, and the top 1 or 5% of genes with the highest connectivity tended to be hub genes. The networks were visualized using Cytoscape_3.3.0. For identification of the genes in each module, GO enrichment pathway analyses were conducted to analyze the biological functions of the modules, and a *q*-value of <0.05 was used as the threshold after correction.

MapMan figures were obtained by running the Mercator tool (http://mapman.gabipd.org/web/guest/mercator, accessed on 18 May 2022) with default parameters to assign MapMan bins to melon transcripts [63]. The Log2 fold changes as obtained from the DESeq output were used as the MapMan input to represent expression changes. The Bioconductor package Pathview version 1.6.0 [64] and Pathview parameters were set as default ones, and the limit parameters were set as follows: limit = list (gene = 5, cpd = 1). As per Pathview default settings, log2-fc values for boxes representing more than one gene were summed. 

We further explored the key hub genes that had important biological functions in each module. First, the original WGCNA output of this study was divided into different modules, which are described in Section 2.4, and modules with significantly high correlation values and where *p* < 0.05 were identified. Subsequently, we defined key hub genes as highly connected and differentially expressed in each significant “black” network module. We then analyzed the hub genes for each heat-map module and visualized them in Cytoscape.

4.4. qRT-PCR Analysis

First strand complementary DNA (cDNA) was synthesized from 100 ng of total isolated RNA using the TRUEscript 1st Stand cDNA Synthesis Kit (Aidlab, Beijing, China) [65]. The gene primers were designed using Primer Premier 6.0 software, and relative expression levels of 5 key genes of the chlorophyll synthesis pathway were analyzed, and transcriptome data between MR-1 and M4-7 was validated using qRT-PCR. The assays were performed in 10 uL of reaction mixture of the SYBR Green I Master Mix, including 20 ng of cDNA and 300 nM of each gene primer. Three biological replicates of each stigma tissue sample and at least three technical replicates of each biological replicate were used for the subsequent expression analysis of five key genes. The transcript level of each key gene was normalized for each sample using the most suitable internal control gene (Actin, *MELO3C023264*), and fold changes in relative gene expression was calculated using the 2^−^^ΔΔ^^CT^ (-delta delta CT) method.

## 5. Conclusions

In this study, comparative transcriptome analysis of the green and yellow stigmas of two different melon lines (MR-1 and M4-7) was performed. The primary differentiation in the chloroplast development of the contrasted stigmas provide morphological support for endogenous pigment content variations. The WGCNA analysis identified a “black” module that was found to be highly associated with stigma color and enriched in photosynthesis, light reactions, light harvesting, the chlorophyll metabolic process, and the chlorophyll biosynthetic process. Meanwhile, the relative expression of five key genes involved in the chlorophyll synthesis pathway was consistently higher in MR-1 (green stigma) than in M4-7 (yellow stigma). These findings evidently suggest that the chlorophyll and its related biological process played an important role in the formation of different colors in melon stigmas. Thus, we believe that our research findings will deliver valuable insights on the in-depth molecular mechanisms of stigma color formation in melons.

## Figures and Tables

**Table 1:** GO enrichment analysis of DEGs in the contrasted melon lines during the stages of stigma development.

GO ID	Classification	Description
Go:0009658	biological process	chloroplast organization
Go:1905392	biological process	plant organ morphogenesis
Go:0010817	biological process	regulation of hormone levels
Go:0042445	biological process	hormone metabolic process
Go:0051640	biological process	organelle localization
Go:0042254	biological process	ribosome biogenesis
Go:0022613	biological process	ribonucleoprotein complex biogenesis
Go:0005618	cellular component	cell wall
Go:0031976	cellular component	plastid thylakoid
Go:0034357	cellular component	photosynthetic membrane
Go:0009570	cellular component	chloroplast stroma
Go:0003735	molecular function	structural constituent of ribosome
Go:0016168	molecular function	chlorophyll binding
Go:0016597	molecular function	amino acid binding

**Table 2:** The gene annotation of 10 hub genes.

Gene_ID	Gene Annotation
*MELO3C014477*	Cyclic nucleotide-gated ion channel 1-like isoform X3
*MELO3C006751*	DNA-binding protein
*MELO3C012015*	WAT1-related protein
*MELO3C011657*	Tetrapyrrole-binding protein, chloroplastic
*MELO3C023604*	Glutathione transport system permease gsiD
*MELO3C014648*	Plastid lipid-associated protein
*MELO3C021788*	Photosystem I reaction center subunit III
*MELO3C006053*	Protein CURVATURE THYLAKOID 1A, chloroplastic
*MELO3C009659*	WD-repeat protein, putative
*MELO3C015140*	Cytochrome P450

## Data Availability

The original contributions presented in the study are included in the article/Supplementary Material, further inquiries can be directed to the corresponding author/s.

## Supplementary Materials

The following supporting information can be downloaded at: https://www.mdpi.com/article/10.3390/ijms23126721/s1.

## Abbreviations

FPKM	Fragments per kilobase of exon model per million mapped fragments
FDR	False discovery rate
DEGs	Differentially expressed genes
GO	Gene ontology
WGCNA	Co-expression network analysis
PSI	Photosystem I
CUTRT1	Curvature thylakoid 1
