# Supplementary material for: Comparative Transcriptome Analysis Identified Key Pathways and Genes Regulating Differentiated Stigma Color in Melon (Cucumis melo L.)
Source: Int J Mol Sci. 2022 Jun 16;23(12):6721. doi: 10.3390/ijms23126721 (PMC9224399; doi:10.3390/ijms23126721)
Supplement: Supplementary file 1 [file ijms-23-06721-s001.zip › ijms-1755499-supplementary.pdf]

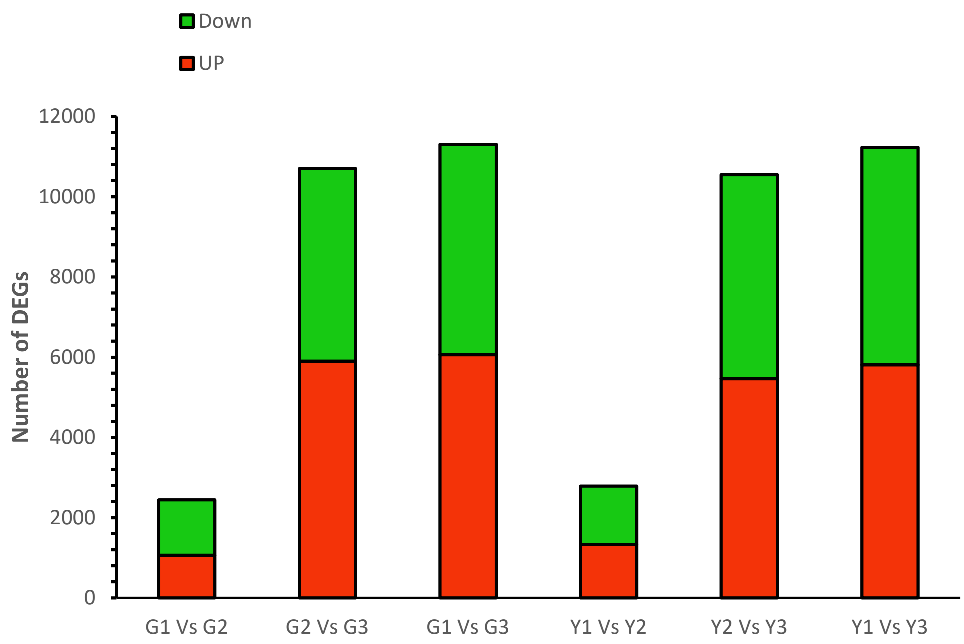

Supplementary Figure S1: The number of differentially expressed genes (DEGs) in MR-1 and M4-7 at each stages of stigma development

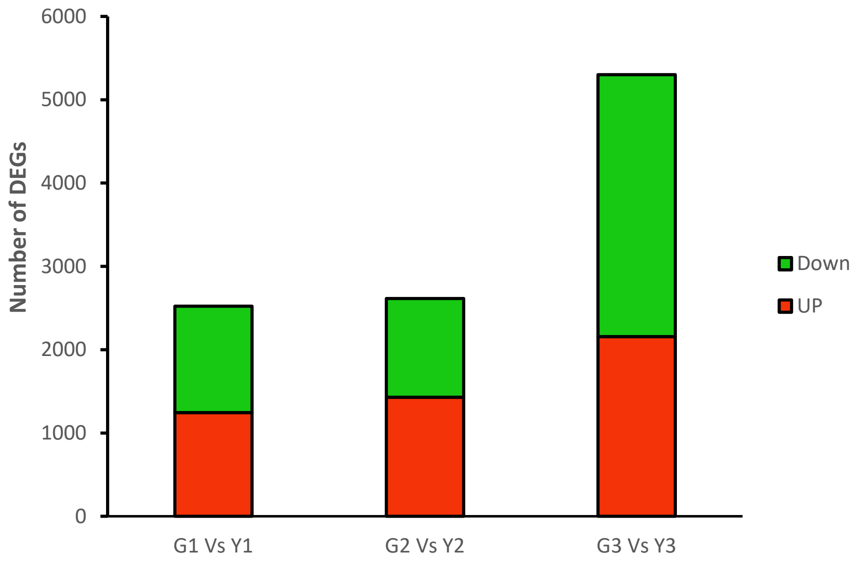

Supplementary Figure S2: The number of differentially expressed genes (DEGs) in MR-1 and M4-7 at same stage of stigma development
